# Disease Progression and Associations of Microvascular Complications in Diabetic Patients: A Study From South India

**DOI:** 10.7759/cureus.30201

**Published:** 2022-10-11

**Authors:** Jayashankar CA, Venkata Bharat Kumar Pinnelli, Darasani Nikethan, Veena Ramachandran, Valakunja H Ganaraja, Shalini A S, Spandana S P, Sravanthi Dandu, Debalina Sarkar

**Affiliations:** 1 Internal Medicine, Vydehi Institute of Medical Sciences and Research Centre, Bangalore, IND; 2 Biochemistry, Vydehi Institute of Medical Sciences and Research Centre, Bangalore, IND; 3 General Medicine, Vishwabharathi Medical College, Kurnool, IND; 4 Rheumatology, Sparsh Hospital, Bangalore, IND; 5 Neurology, Vydehi Institute of Medical Sciences and Research Centre, Bangalore, IND; 6 General Medicine, Vydehi Institute of Medical Sciences and Research Centre, Bengaluru, IND; 7 General Medicine, Vydehi Institute of Medical Sciences and Research Centre, Bangalore, IND

**Keywords:** diabetic microvascular complications, oral hypoglycemic agents, types 2 diabetes, diabetic nephropathy (dn), diabetic retinopathy

## Abstract

Diabetes mellitus (DM) is a chronic metabolic disease characterized by inappropriately elevated blood glucose levels. If not treated at the early stage, it can lead to complications like diabetic retinopathy (DR) and diabetic nephropathy (DN) which are often associated with severe morbidity and mortality. This study was designed to identify the prevalence of retinopathy and nephropathy in diabetic patients and also to determine the correlation between DR and DN. In this cross-sectional study, a total of 84 diabetic patients (Male: Female- 53:31) were included. The mean age at presentation was 54.06 ± 9.85 years. Among them, 28% of patients had a duration of diabetes of < 5 years. Nearly 42% and 30% of patients had diabetes between 5-10 years, and more than 10 years respectively. At the time of presentation to us, a total of 42.8% of patients had a combination of nephropathy and retinopathy, 40.4% of patients had only retinopathy, and 16.6% of patients with only nephropathy. Among patients with nephropathy and microalbuminuria, only 5.9% had DR ranging from mild to a moderate degree and none had severe DR. In patients with macroalbuminuria, 26.2% had moderate to severe DR. Microvascular complications are more prevalent in diabetics with disease progression. Microalbuminuria is a marker for retinopathy and these patients require ophthalmic evaluation at the earliest. Early recognition and management of these, can reduce the occurrence of complications as well as disease progression, thus reducing the related mortality.

## Introduction

The term ‘Diabetes Mellitus’ (DM) is derived from the Greek word 'Diabetes' which means siphon to pass through and a Latin word 'Mellitus' meaning sweet [1]. It is a metabolic disease characterized by inappropriately elevated blood glucose levels [1]. Globally, the total number of people with diabetes is expected to rise from 171 million in 2000 to 366 million in 2030 [2].

This metabolic dysregulation in diabetes also causes secondary pathophysiologic changes in multiple organ systems resulting in chronic vascular and non-vascular complications. Major complications of DM are microvascular complications such as retinopathy, neuropathy, and nephropathy which can cause severe morbidity if neglected [3]. This study was designed to identify the prevalence of retinopathy and nephropathy in type Ⅱ diabetics. Secondary outcomes were included to determine the correlation between DN and DR in these patients.

## Materials and methods

This is a cross-sectional study done by convenience sampling technique in patients aged 18 and more years with type Ⅱ diabetes who were attending the outpatient departments of the General Medicine department attached to a tertiary care teaching hospital in southern India from January 2013 to December 2013.

Diabetic patients with other associated conditions altering fundus examination findings like, those who have had ocular procedures or surgeries in the past, patients with hypertension, congestive cardiac failure, active urinary tract infection, and pregnant diabetics were excluded from the study. This study was approved by the Institute Ethics committee of Vydehi Institute of Medical Sciences and Research centre with No: VIMS/IEC/PGThesis/2012.

Case definitions

Patients were considered to be having nephropathy if they have microalbuminuria or overt proteinuria based on a positive albustix test in ≥2 consecutive urine samples. A 24-hour urine sample was collected from 8 am to 8 am and was subjected to an automated urine protein analyser which employs a turbidimetric method to quantify proteinuria. DN was graded as microalbuminuria (30-300mg/24 hours), macroalbuminuria (300-3000mg/24 hours), and massive proteinuria (>3000mg/24 hour).

Patients were labelled to be having DR on the basis of fundoscopic and ocular examination done by physicians which included best-corrected visual acuity (BCVA) using Snellen‘s charts, colour vision, dilated fundus evaluation using direct/indirect ophthalmoscope, and slit lamp. DR was defined and graded based on the Early Treatment of Diabetic Retinopathy Study (ETDRS) classification into Mild, Moderate, Severe, and Very severe non-proliferative diabetic retinopathy (NPDR) and Proliferative diabetic retinopathy (PDR) with or without high-risk characteristics [4]. Diabetic macular oedema was categorized into Clinically Significant Macular Edema (CSME) and non-CSME. 

Blood investigations such as fasting blood sugar (FBS), postprandial blood sugar (PPBS) & Glycosylated haemoglobin (HbA1c), blood urea, and serum creatinine were measured for all patients.

Statistical analysis

The quantitative data were represented as mean, median, interquartile range, and standard deviation (SD). Frequency percentage was used for categorical variables.

## Results

A total of 84 diabetic patients (male: female=53:31) were included in the study. The mean age at presentation of these patients was 54.06 ± 9.85 years (31-90 years). Among them, 28% of patients had a duration of diabetes < 5 years and 42% had a duration of diabetes between 5-10 years, and the remaining 30% had diabetes for more than 10 years. Among these diabetic patients, 80.9% were on oral hypoglycaemic agents (OHA) and 19.1% were on insulin therapy. Approximately 16.6% of patients had only nephropathy and 40.4% of the patients had only retinopathy. The remaining 42.8% of patients had a combination of both. Among patients with retinopathy, 38.1% of the patients had DR with diabetes mellitus with a duration of diabetes less than 5 years, 25% of patients had retinopathy with a duration of diabetes between 5 to 10 years, and 20.2% of the patients had DR with a duration of diabetes more than 10 years. The number of patients with moderate NPDR, severe NPDR and PDR increased as the duration of DM increased (Table 1, 2).

**Table 1:** Clinical features and laboratory parameters of Diabetic Retinopathy (DR) patients and its subgroups. dL- decilitre; HbA1C- glycated haemoglobin; IQR- interquartile range; Max- Maximum; mg- milligram; Min- Minimum; n- number of patients; NPDR- non-proliferative diabetic retinopathy; SD- standard deviation.

Variables		Total (n=84)	Normal(n=14)	Mild NPDR(n=37)	Moderate NPDR(n=19)	Severe NPDR(n=10)
Age (Years)	Mean ± SD	54.06 ± 9.85	50.05 ± 8.00	50.49 ± 8.33	57.42 ± 8.10	62.90 ± 13.14
	Min-Max	31-90	35-65	31.00-72.00	44.00-75.00	4.00-90.00
	Median (IQR)	54(48.74-58.25)	51.5(45-56.75)	51(47.00-55.00)	57(52.00-61.50)	60.5(55.00-65.75)
Gender	Female	31(36.9%)	3(21.43%)	12(32.43%)	10(52.63%)	5(50%)
	Male	53(63.1%)	11(78.57%)	25(67.57%)	9(47.37%)	5(50%)
Duration of Diabetes (Years)	Mean ± SD	7.78 ± 6.36	5.5 ± 2.71	4.66 ± 3.68	9.58 ± 5.96	16.9 ± 8.19
	Min-Max	44591	1.00-10.00	1.00-20.00	4.00-30.00	5.00-28.00
	Median (IQR)	6(4-10)	5.50(3.50-7.75)	4(3.00-6.00)	8(6.00-10.50)	17.00(12.00-23.75)
Treatment received	Insulin	16(19.05%)	2(14.29%)	2(5.41%)	3(15.79%)	7(70%)
	OHA	68(80.95%)	12(85.71%)	35(94.59%)	16(84.21%)	3(30%)
HbA_1_c	Mean ± SD	8.96 ± 6.20	7.814 ± 1.58	8.9 ± 9.14	8.87 ± 1.71	10.02 ± 2.29
	Min - Max	5.00-16.2	5.80-11.7	5.00-8.00	6.30-13.40	7.500-14.00
	Median (IQR)	8(7-9)	7.5(6.85-8.45)	7.00(6.80-8.00)	8.50(8.00-9.55)	9.40(11.6514.00)
24-hour urine albumin (mg)	Mean ± SD	762.4 ± 1068.07	989.6 ± 1182.24	79.3 ± 181.86	1185.9 ± 1174.21	1926 ± 1135.80
	Min - Max	0-3705	56.0 -3122.0	0.00-790.0	0-3443.00	567.00-3705.00
	Median (IQR)	159(0-1349.5)	174.0 (134.2-1859.5)	6.69	980.0(222.50-1734.50)	1818.00(887.0-2865.00)
Blood urea (mg/dL)	Mean ± SD	36 ± 15.49	41.29 ± 25.62	30.00 ± 6.69	36.5 ± 10.63	47.28 ± 14.3
	Min-Max	15.0-98.0	15.0-98.0	16.0-62.0	20.5-61.0	27.0-67.0
	Median (IQR)	31(28-37)	33.00(28.2-43.00)	30(28-31)	34.0(31.0-37.50)	45.00(37.25-61.25)
Serum creatinine (mg/dL)	Mean ± SD	1.94 ± 1.99	2.12 ± 1.31	0.98 ± 0.36	2.72 ± 2.4	3.01 ± 2.39
	Min - Max	0.41-10	0.50-4.60	0.41-2.18	0.80-9.90	1.10-9.30
	Median (IQR)	1.20(0.90-2.10)	2.10(1.13-2.40)	0.92(0.73-1.10)	1.54(1.10-3.14)	2.20(1.88-2.98)

**Table 2:** Table showing duration of Diabetes Mellitus (DM) and Diabetic Retinopathy (DR) DR: diabetic retinopathy; NPDR: non-proliferative diabetic retinopathy; PDR: proliferative diabetic retinopathy.

Duration of diabetes mellitus	Grade of diabetic retinopathy					Total
	No DR (%)	Mild NPDR (%)	Moderate NPDR (%)	Severe NPDR (%)	PDR (%)	
<5 years	7(8.3%)	26(30.1%)	5(5.9%)	1(1.2%)	0	39(46.4%)
5-10 years	7(8.3%)	8(9.5%)	10(11.9%)	1(1.2%)	2(2.4%)	28(33.3%)
>10 years	0	2(2.4%)	5(5.9%)	8(9.5%)	2(2.4%)	17(20.2%)
Total	14(16.6%)	36(42.8%)	20(23.8%)	10(11.9%)	4(4.8%)	84(100%)

Among the patients in this study group, a majority of patients had macroalbuminuria (34.5%), nearly 15.5% had microalbuminuria, and 9.5% had massive proteinuria. In patients with microalbuminuria, 9.5% did not have DR, 5.9% had DR ranging from mild to a moderate degree, and none had severe DR. In the group with macroalbuminuria, only 4.7% did not have DR, whereas a majority of patients (26.2%) had moderate to severe DR. Among the patients with massive albuminuria, 2.4% did not have DR, while 7.1% had moderate to severe DR. The relationship between DR and DN is shown in table 3.

**Table 3:** Table showing relation between Diabetic Retinopathy (DR) and Diabetic Nephropathy (DN). DR: diabetic retinopathy; NPDR: non-proliferative diabetic retinopathy; PDR: proliferative diabetic retinopathy; mg: milligram.

Urine protein (mg/24-hour)	Grade of diabetic retinopathy					Total (%)
	No DR	Mild NPDR	Moderate NPDR	Severe NPDR	PDR	
No albuminuria	0	28(33.3%	5(5.9%)	0	1(1.2%)	34(40.4%)
30-300 microalbuminuria	8(9.5%)	3(3.6%)	2(2.4%)	0	0	13(15.4%)
300-3000 macro albuminuria/ proteinuria	4(4.8%)	5(5.9%)	9(10.7%)	8(9.5%)	3(3.6%)	29(34.5%)
>3000 massive albuminuria/proteinuria	2(2.4%)	0	3(3.6%)	3(3.6%)	0	8(9.5%)
Total	14(16.6%)	36(42.8%)	19(22.6%)	11(13.1%)	4(4.7%)	84(100%)

## Discussion

We hereby describe our experience with microvascular complications of DM and the features of disease progression with time. Studies that looked at the incidence of DR and the association between DN and DR are very limited in India. Hence, this study was done to identify the prevalence and association of DN and DR in our study population. The increasing prevalence and longer duration of diabetes is more likely to alter the disease profile in many diabetic patients, especially with a higher incidence of microvascular complications [5].

One of the most important complications of DM is DN, which is characterized by the triad of hypertension, proteinuria, and renal impairment. To date, DN remains a major cause of morbidity and mortality in diabetics and is prevalent in 30-35% of diabetic patients globally and is associated with a 2-4 fold increased risk of death in comparison to the general population [1,5]. Similarly, DR is also a leading cause of acquired blindness, and the presence of DR may independently predict the progression of diabetic nephropathy by predicting the development of microalbuminuria [6]. There is a direct correlation between the frequency and severity of DR with respect to the duration of DM [7].

In a meta-analysis done in 2013 with 26 studies, it was found that PDR was a more specific predictor of DN [7]. Conversely, it would be useful to know if the presence of DN would help in predicting progression to DR. Previously, the term ‘retinal-renal syndrome’ was coined with the aim to define a group of high-risk patients with coincident kidney and eye diseases due to diabetes [8]. The main pathophysiology in both these clinical conditions is due to diabetic microvasculopathy in retinal and glomerular arterioles and capillaries, which explains the overlap of these two clinical conditions in diabetic patients [8].

Usually, DR precedes DN. However, the converse may also be possible. As DN is microvascular in origin which starts with the thickening of the glomerular basement membrane early in diabetic renal disease, this results in nodular, diffuse, and exudative glomerular lesions leading to renal glomerular hyalinization lesions. This is primarily an ischemic event and shares similar risk factors including prolonged duration of diabetes and poor glycaemic control [8].

In our study, it was also observed that the presence of proteinuria was more in patients with retinopathy and was directly related to the severity of nephropathy. Similarly, in the Chennai Urban Rural Epidemiology Study (CURES), proteinuria was observed in nearly one-third of the patients with DR [9]. Even studies from North India also have suggested a similar relationship between DR and microalbuminuria [10]. The prevalence of DR in our study group was higher in patients with macroproteinuria as compared to those with microproteinuria, thereby, highlighting the importance of early detection and treatment of nephropathy. This could reduce the mortality of such patients from the other complications of nephropathy.

Many epidemiological studies done previously support the role of DN as one of the risk factors for the development of DR suggesting that DN patients benefit from having a regular ophthalmic evaluation. Conversely, the presence of retinopathy is also a risk indicator of DN and these patients may benefit from screening for 24-hour urine protein estimation. Early detection of DN and its treatment could reduce morbidity and mortality of such patients from other complications of DN and DR [11].

In our study, there was a unidirectional correlation association between DR and DN which can be explained by chronological order, that is DN precedes DR. It also indicates that the level of renal impairment is proportional to the level of damage to the eye similar to studies observed before [11,12]. All patients with proteinuria and diabetic retinopathy have diabetic nephropathy. In this study, PDR was present in patients with macroalbuminuria. Similar results were also observed in other previous studies [6,12].

A major limitation of this study was as only type Ⅱ diabetics were included, the other types of DM were not studied. However Intensive diabetic control leads to a reduction in the development and progression of all diabetic complications.

## Conclusions

In the present study, there is a direct correlation between DN and DR. Microalbuminuria or macroproteinuria is a marker for retinopathy and these patients require ophthalmic evaluation at the earliest. It is recommended to screen all newly detected diabetic patients for diabetic retinopathy and microalbuminuria. Also, regular follow-ups are advised with increasing duration of diabetes. A good glycaemic control and regular follow-ups can reduce the occurrence as well as the progression of the disease, thus reducing the related mortality and morbidity.
